# Effect of Physical Exercise Program Based on Active Breaks on Physical Fitness and Vigilance Performance

**DOI:** 10.3390/biology10111151

**Published:** 2021-11-08

**Authors:** Francisco Tomás González-Fernández, Sixto González-Víllora, Salvador Baena-Morales, Juan Carlos Pastor-Vicedo, Filipe Manuel Clemente, Georgian Badicu, Eugenia Murawska-Ciałowicz

**Affiliations:** 1 Department of Physical Activity and Sports Sciences, Pontifical University of Comillas (Centro de Estudios Superiores Alberta Giménez), 07013 Palma de Mallorca, Spain; francis.gonzalez.fernandez@gmail.com; 2 SER Research Group, Pontifical University of Comillas (Centro de Estudios Superiores Alberta Giménez), 07013 Palma, Spain; 3 Department of Physical Education, Arts and Music, University of Castilla-La Mancha, 02006 Albacete, Spain; sixto.gonzalez@uclm.es (S.G.-V.); juancarlos.pastor@uclm.es (J.C.P.-V.); 4 EDUCAFD Research Group, Department of General Didactic and Specific Didactics, Faculty of Education, University of Alicante, 03690 Alicante, Spain; salvador.baena@ua.es; 5 Escola Superior Desporto e Lazer, Instituto Politécnico de Viana do Castelo, Rua Escola Industrial e Comercial de Nun’Álvares, 4900-347 Viana do Castelo, Portugal; filipe.clemente5@gmail.com; 6 Instituto de Telecomunicações, Delegação da Covilhã, 1049-001 Lisboa, Portugal; 7 Department of Physical Education and Special Motricity, University Transilvania of Brasov, 500068 Brasov, Romania; georgian.badicu@unitbv.ro; 8 Department of Physiology and Biochemistry, University School of Physical Education, 51-612 Wrocław, Poland

**Keywords:** physical activity, executive functions, cognitive performance, youth, physical education

## Abstract

**Simple Summary:**

Our study aimed to analyze the effects of 8 weeks physical training on vigilance performance in high school students. Forty-two healthy students were assigned for convenience and matched into two groups, a Control Group (CG) and an Active-Break Group (ABG). The participants were assessed before the training program using the Alpha-Fitness test battery and Psychomotor Vigilance Task (PVT) to observe their physical fitness and vigilance performance. Compared with the pre-test, significant different were observed in the post-test PVT. Results showed a main effect of ABG responding faster than students in the CG group. This demonstrated that 8 weeks physical training have an effect on vigilance performance and improve the efficiency of vigilance in high school students.

**Abstract:**

The scientific literature has shown the beneficial effects of chronic Physical Exercise (PE) on a wide range of tasks that involve high-order functioning. For this reason, the present study aimed to investigate the effects of active breaks on physical fitness and vigilance performance in high school students through eight weeks of physical training. A total of 42 healthy students (age = 16.50 ± 0.59 years; height = 171.08 ± 8.07 cm; weight = 67.10 ± 13.76 kg) from one Andalusian high school (Spain) were assigned for convenience and matched into two groups, a Control Group (CG) and an Active-Break Group (ABG). The ABG performed two active breaks (based on strength and self-loading exercises) during the school day, first at 10:00 a.m. and second at 12:30 p.m. The participants were assessed before and after the training program using the Alpha-Fitness test battery and the Psychomotor Vigilance Task (PVT). Significant differences were observed in the post-training PVT results, compared with the pretraining PVT, showing ABG responding faster than CG. Thus, the presents study demonstrated that eight weeks of physical training affects vigilance performance (compared to CG) and improves the efficiency of vigilance in high school students, contributing to enhancement of quality of education.

## 1. Introduction

In the last decade, there has been a rapidly growing interest in the scientific knowledge that links chronic physical exercise (PE) and cognitive performance [1,2,3,4,5]. A comprehensive review of the scientific literature has shown the beneficial effects of chronic PE on a wide range of tasks involving high-order functioning, such as attention, cognitive control, memory, and perception, among others [6]. The vast majority of the studies in this field have focused on the impact of chronic PE on executive functions [5,7,8], and to a lesser extent, on tasks that involve short-term memory [9,10], attention [11], and language processing [12]. However, current research has shown that regular PE produces different constant changes, such as those at the structural level involving angiogenesis or neurogenesis in different areas of the brain, especially in the hippocampus [13,14]. There is also an increase in blood vessels in the hippocampus, cortex, and cerebellum, which increase the supply of nutrients and energy in these neural areas [15]. It has been widely demonstrated that performing regular exercise at moderate aerobic intensities (40% to 80% of maximum oxygen consumption (VO_2máx_)) acts positively on cognitive tasks such as processing speed, selective attention, and short-term memory [3,5]. Finally, there is an increase in brain structures due to neuronal plasticity, increased vascularization, and neurogenesis (brain plasticity). The evidence suggests that these adaptations produce a better cognitive response in various tasks, including memory, attention, processing speed, cognitive flexibility, and inhibition.

Vigilance refers to the cognitive (attentional) function that determines the capacity to respond appropriately (quickly and accurately) to relevant stimuli [16]. In the laboratory, vigilance is typically investigated using tasks involving the monotonous presentation of stimuli for a relatively long period of time, requiring participants to detect rare events [17] or to simply respond to unpredictable target onsets [18]. Low levels of vigilance result in slow reaction time (RT), response anticipation, or even failure to detect the target. Consistent findings in sustained attention research show a decline in performance with time-on-task, the so-called vigilance decrement. Researchers have suggested that this performance decrement over time reflects a decrease in attentional resources [19,20,21]. A cursory look at the literature reveals studies investigating vigilance primarily in the context of various everyday activities [22,23]. However, scientific research on the relationship between regular exercise (based on ABs) and vigilance in the high school setting is lacking. In this respect, ABs have been applied in classrooms using different motor games and including varied coordination abilities, locomotor skills (e.g., running, jumping, or sliding), and stability skills (e.g., balance, bending, or turning). Moreover, the results of previous research obtained after physical interventions have found a positive relationship between physical fitness acquired on intervention and cognitive performance [24,25,26]. Therefore, we suggest that vigilance could be exponentially affected during the school day, and more specifically, that ABs could contribute positively to the vigilance performance, both immediately after ABs as well as chronically, i.e., with a long-lasting effect caused by the chronic implementation of ABs. This may be significant, especially in the academic context, given the importance of vigilance for maintaining optimal performance during the course of the school day [27].

The scientific literature reported to date points to the positive influence of physical fitness on vigilance [28,29,30]. A small number of studies carried out have been conducted at early ages (i.e., in children from 3 to 12 years old) and show a positive relationship between the level of physical fitness and vigilance. For example, Pontifex et al.’s [31] study examined the performance of vigilance as a function of time-on-task, using an Eriksen flanker task in two age groups (preadolescents with a low level of physical fitness and preadolescents with a high level of physical fitness). Their results showed an increase in the rate of omission error and the number of sequential omissions as a function of time-on-task in preadolescents with low physical fitness. Another study investigated the time course of behavioral performance and brain functioning in preadolescents with high and low levels of physical fitness [31]. The study found a decrease in performance reflected in the incongruent trials in the low physical fitness group, evidenced by an increase in bilateral activation of frontal and parietal brain regions. In contrast, participants with high physical fitness showed a decrease in physical activity as a function of time-on-task, although in the initial time block, they showed better activity compared with participants with low physical fitness. Bunce [28] carried out research with young and older adults that attempted to analyze the performance of vigilance according to a degree of complexity of the task and a level of physical fitness. Results indicated a smaller decrease in vigilance in the group of older adults with a high level of physical fitness than in the low-level physical activity group. These results are reflected in tasks with high demands on attention resources, e.g., when information or complexity is high.

In summary, the existing literature confirms the important role that PE and the level of physical fitness [10,32,33] play in cognitive performance in areas involving vigilance. However, research on this is still scarce, and a number of questions still need to be answered. The main purpose of this research was to examine the chronic effects of eight weeks of physical training on cognition, specifically on vigilance performance, in high school students. It should be noted that, based on the evidence found in the literature on regular PE and vigilance, most studies are limited insofar as they show experimental designs between groups and can only look for relationships between vigilance and physical condition indirectly. The central hypothesis of the current study is that an 8-week physical training based on AB would enhance physical fitness and vigilance performance, and in doing so, draw a direct link between cognition and PE.

## 2. Materials and Methods

### 2.1. Study Design

The study was conducted between January and February of 2020. At the time of these observations, the students had completed three months of training and familiarization with training protocols, tasks, and rating of perceived Exertion (RPE) during Physical Education class. A quasi-experimental pre–post design control group (CG) and an active break group (ABG) were used in the present research. The high school students were selected for convenience, assigned and matched into two groups, an ABG and a CG, based on the class to which they belonged. To investigate the effects of an 8-week AB-based Physical Exercise Program on physical fitness and vigilance performance, those in the CG were asked to maintain their ordinary routines and training practices, while those in the ABG modified their training sessions by introducing ten minutes of AB before 10:00 a.m. and another AB before 12:30 p.m.

### 2.2. Participants

A total of 42 healthy students from a high school in the region of Andalusia, Spain, participated in this study: 25 girls (age = 16.42 ± 0.50 years; height = 167.66 ± 6.30 cm; weight = 64.13 ± 11.78 kg) and 17 boys (age = 16.62 ± 0.71 years; height = 176.53 ± 7.73 cm; weight = 72.06 ± 15.58 kg) as shown in Table 1. Concerning the sample size, the next equation was used: *Sample Size* = *Z*2 × (*p*) × (1 − *p*)/*C2*, where Z = confidence level (95%); *p* = 0.05 and *C* = margin of error 0.05. Participants were recruited from the city of Granada, which has a population in the range from 100,000 to 1,000,000 inhabitants according to the National Institute of Statistics from the Spanish Government (http://www.ine.es/ accessed on 1 March 2021).

Inclusion criteria for the participants in this study were: (i) reporting normal vision and no history of any neuropsychological impairments that could affect the results of the experiment, (ii) not presenting any injuries during the previous two months, (iii) giving consent, and (iv) participating in 85% of the AB during the study period.

In addition, all participants completed a healthy lifestyle questionnaire and the short version of the international physical activity questionnaire (IPAQ-SF) [35]. In the first document, they were asked about current sports habits, addictions, and diseases that could impede the practice of physical exercise, while, in the second, we recorded their level of physical activity (PA).

The participants were informed about the main goals of the investigation and signed informed consent forms. Families were informed that they could revoke the participation agreement at any time. The students were treated according to the American Psychological Association (APA) guidelines, which ensured the anonymity of participants’ responses. In addition, the study was conducted following the ethical principles of the 1964 Helsinki declaration for human research and was approved by the Research Ethics Committee of the University of Castilla-La Mancha (Hospital Universitario de Albacete, Record April 2020, and internal project n° 2020/05/052).

### 2.3. Procedure

#### 2.3.1. Preintervention

First, the school management team was informed about the study objectives and ensured that the students’ parents or guardians had signed informed consent forms detailing the possible benefits and risks. Subsequently, the study and the plan for the structure of every class day were designed together with the school’s teachers and advisors. In order for the students not to miss any classes, we use the ABs to improve performance in the classroom. Every task or a test was monitored by one lead researcher assisted by a physical education teacher responsible for the groups, specially trained for accurate and reliable data recording, especially in Physical Fitness assessment.

In the first session, participants filled out the questionnaires (≈10 min to assess the entire group), had the anthropometry assessment (≈15 min to assess the entire group), and tests from the ALPHA-Fitness test battery in the following order: 4 × 10 m speed-agility (≈10 min to assess the entire group) and standing broad jump (≈15 min to assess the entire group). Participants were given adequate time to fully recover due to the neuromuscular characterization of the different tests applied in this session. In addition, the application order that the students followed during the research was always the same. Therefore, the measures taken ensured the recovery. In the second session, participants performed the 20 m shuttle run test (≈30 min to assess the entire group) see Section 2.4. Finally, in the third session, students performed the PVT to determine their basal level of vigilance.

All measures were recorded at the same time of day, between 10:30 a.m. and 14:30 p.m., in the same space and time with the same humidity conditions (30–40%). The students were familiarized with the use of the subjective rating of perceived exertion scale (RPE) during their physical education classes. In addition, the ABG group was instructed in an 8-week PE program based on ABs in the physical education class for three months. Every task or a test was monitored by one lead researcher assisted by a physical education teacher responsible for the groups specially trained for accurate and reliable data recording, especially in physical fitness assessment.

#### 2.3.2. Intervention

**Figure 1:**
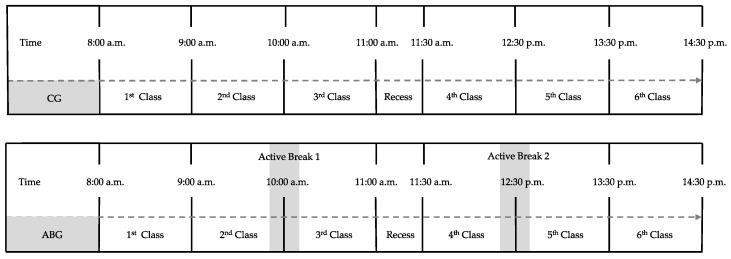
Schematic representation of the school day procedure of one session of the experimental group.

The students completed eight weeks of training based on functional training. In the case of ABG, they performed two ABs (based on strength and self-loading exercises) during the school day, AB1 at 10:00 a.m. and AB2 at 12:30 p.m. (Figure 1). The training program was supervised by the lead researcher and teachers responsible for the ABG and was designed individually for each participant, considering their individual physical characteristics (each exercise was designed with an adaptation in difficulty and intensity). We followed the guidelines of the American College of Sports Medicine [36] to ensure the safety of the participants.

**Figure 2:**
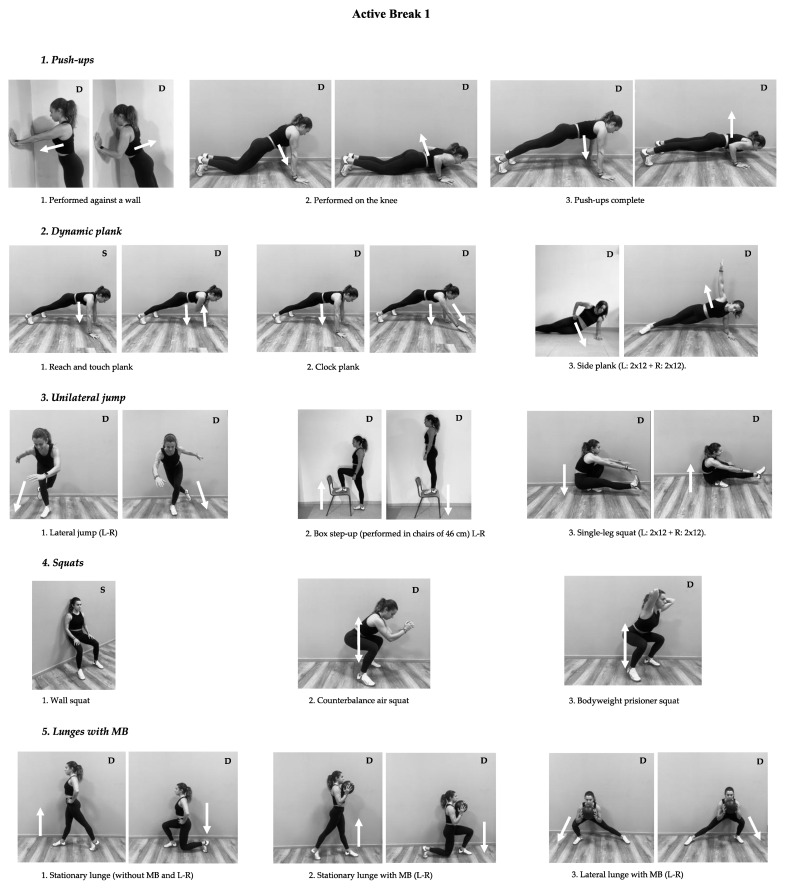
Description of Active Break 1 for high school students. (Arrows represent the movement direction, and S was used for static and D for dynamic movement).

**Figure 3:**
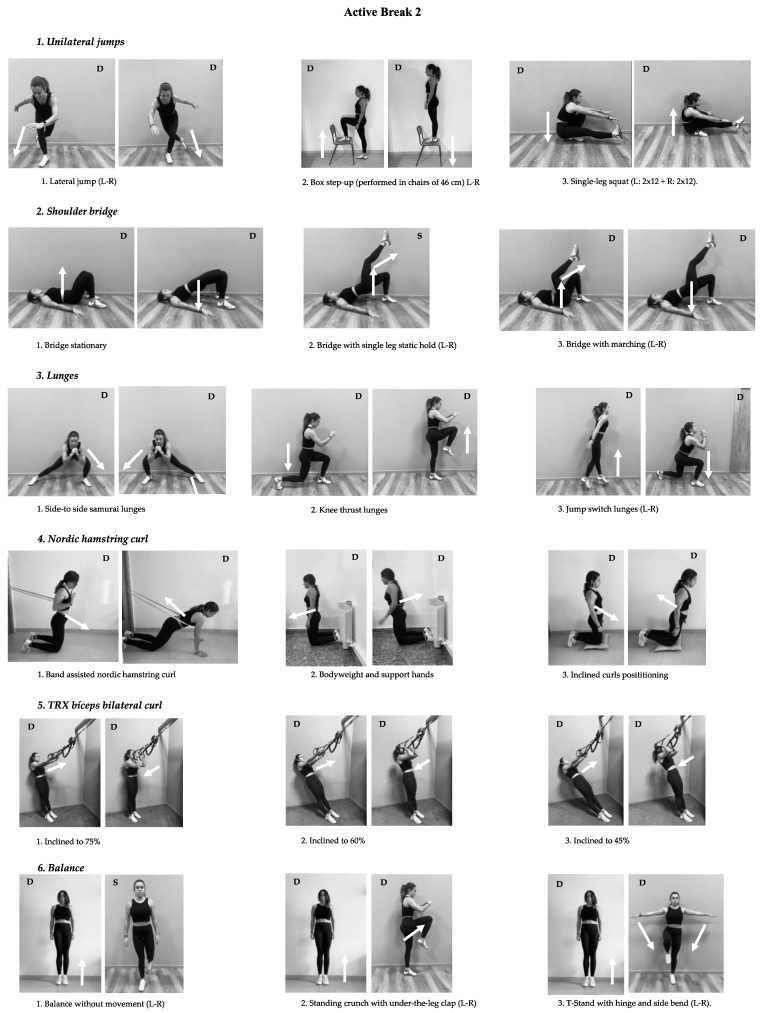
Description of Active Break 2 on high school students. (Arrows represent the movement direction, and S was used for static and D for dynamic movement).

A total of 80 ABs was performed by each participant. Training sessions in both ABs were divided into three phases: (i) warm-up 2 min (dynamic stretching and joint mobility exercises); (ii) training activity ≈8 min (more information in Figure 2 and Figure 3 ); and (iii) cooldown 2 min (stretching exercises). According to the RPE scale, the average intensity of the sessions was between hard and very hard (16.10 ± 1.12). To control the intensity of the training program, we used a google questionnaire that recorded RPE immediately after each AB. Crucially, the CG were instructed to maintain their classroom routines while the ABG performed the AB. The very light average intensity of sessions (6.29 ± 0.42) was also recorded.

In the training activity, students worked in a circuit. Thus, equipment was distributed around the classroom, and students performed the AB exercises in sequence until finished. The exercises of AB1 (4 sets ×12 sec a recovery time between exercises of 10 sec and between sets of 20 sec) and AB2 (4 sets × 10 sec and a recovery time between exercises of 10 sec and between sets of 20 sec) were the same during the whole intervention. However, the progress of all sessions varied by weeks due to the heterogeneity of the sample and the individuals’ varied physical conditions. All students were familiarized with the exercises and the exercise intensity (between hard and very hard).

Active Break 1 consisted of the following exercises: Push-ups progresssion: (i) performed against a wall, (ii) performed on the knees, and (iii) push-ups complete; Dynamic plank: (i) reach and touch plank; (ii) clock plank, and (iii) side plank (left: 2 × 12 + right: 2 × 12); Unilateral jump: (i) lateral jump (left-right), (ii) box step-up performed in chairs of 46 cm (left-right), and (iii) single-leg squat (left: 2 × 12 + right: 2 × 12); Squats: (i) wall squat, (ii) counterbalance air squat, and (iii) bodyweight prisoner squat; Lunges with MB: (i) stationary lunge (without MB and left-right), (ii) stationary lunge with medicine ball of 4, 6 or 8 kg (left-right), and (iii) lateral lunge with MB (left-right).

Active Break 2 consisted of Unilateral jump: (i) lateral jump (left-right), (ii) Box step-up performed in chairs of 46 cm (left-right), and (iii) single-leg squat (left: 2 × 12 + right: 2 × 12); Shoulder bridge: (i) Bridge stationary, (ii) bridge with single leg static hold (left-Right), and (iii) bridge with marching (left-right). Lunges: (i) side-to-side samurai lunges (Left-right), (ii) Knee thrust lunges (left-right), and (iii) jump switch lunges (left-right); Nordic hamstring curl: (i) band-assisted nordic hamstring curl, (ii) bodyweight and support hands, and (iii) inclined curls positioning; TRX biceps bilateral curl: (i) inclined to 75%, (ii) inclined to 60%, and (iii) inclined to 45%; Balance: (i) balance without movement (left-right), (ii) standing crunch with under-the-leg clap (left-right), and (iii) T-Stand with hinge and side bend (left-right).

See Figure 2 and Figure 3 to see the variations provided to students to adjust the level of difficulty to their ability in order to execute the exercise. 

#### 2.3.3. Post-Intervention

After eight weeks, both groups were evaluated at the same time of day as in the pre-intervention session (between 10:30 a.m. and14:30 p.m.), in the same space, with the same humidity conditions. Everything was the same as in the preintervention session except t that questionnaires and the ALPHA-Fitness test battery were not completed. Notably, after the termination of the study, students from the CG were also given the opportunity to perform the same program as the experimental group.

### 2.4. Measures

#### 2.4.1. Anthropometry

Height and body weight were measured before the start of the intervention. A bioelectrical impedance analysis (BIA) device (Tanita BC-730) was used to calculate body weight (kg) to the nearest 0.1 kg. Both measures were assessed by one main researcher (Pre and Post). A stadiometer (Type SECA 225, Hamburg, Germany) was utilized to measure the height (cm) to the nearest 0.1 cm. Participants were asked to remove their shoes and other accessories that could influence the assessment. They also had to be vertical and immobile, with arms extended along the body and looking straight ahead in an upright position. For each parameter, only one measurement was collected.

#### 2.4.2. Physical Fitness Assessment

The level of physical fitness was assessed using the ALPHA-Fitness test battery [37]. We followed the protocol established for this test battery and the guidelines of the ACSM [36] to ensure the safety of the participants. In addition, to ensure successful performance in the ALPHA-Fitness test battery, all the students were informed about the protocol to ensure an adequate data collecting process in both groups.

##### 20-m Shuttle Run Test

Cardiorespiratory fitness (CRF) was assessed with the 20 m shuttle run test [34]. Students were required to run between two lines separated by 20 m while keeping pace with audio signals emitted from a USB Player with the test protocol. Students started the test with an initial speed of 8.5 km/h^−1^, which was increased by 0.5 km/h^−1^ min^−1^ (stage duration = 1 min). We recorded the last one-half stage completed as an indicator of CRF. In addition, VO2_max_ was estimated with the equation VO2_max_ = 5.857 × velocity (km/h) − 19.45.

##### 4 × 10 m Speed-Agility Test

Coordination, agility, and speed were evaluated with this test. The aim of the test was to run four repetitions of 10 m distance. Students had to run at a maximum speed, and they had two attempts. We recorded the best of the two attempts, and results were measured in seconds with a Casio handheld stopwatch (HS-3V-1).

##### Standing Broad Jump

This test has been successfully used for measuring lower limb explosive strength. Students jumped horizontally to achieve maximum distance (in centimeters). Participants performed the standing broad jump three times, with 20 s of recovery between attempts to minimize the effect of fatigue. The best jump was considered as the final outcome. The test was performed in the school gym to avoid falls caused by slipping [38].

#### 2.4.3. Rating of Perceived Exertion (RPE)

The RPE was measured with the Borg scale [39] immediately after the exercise in ABG and CG. The RPE scale ranged from 6 (no exertion) to 20 (maximal exertion).

#### 2.4.4. Cognitive Measurement: Psychomotor Vigilance Task

iPhones 5s (iOS 12.4.5) were used to present the stimuli of the PVT. Performance in the PVT has been shown to be valid to control vigilance [18,40] and was linked to the control of cardiovascular fitness [41]. The devices were previously blocked to any other type of notification. The center of the mobile screen was placed about 50–80 cm from the participants’ heads at eye level (aiming to help everyone feel as comfortable as possible during the duration of the task). The PVT presents a grey screen with a chronometer at the center, which begins the countdown at the speed of a real stopwatch and could be presented on the screen after a random time interval ranging between 2000 and 10,000 ms. Verbal and written instructions were given to the participant prior to the start of the PVT in every session, stressing that they had to fixate on the center of the screen, try not to move their eyes, and respond as quickly as possible (while avoiding anticipation errors) as soon as the chronometer starts. The task included a single block lasting 10 min. The exact number of trials of each participant depended on the latency of the individual’s response.

**Figure 4:**
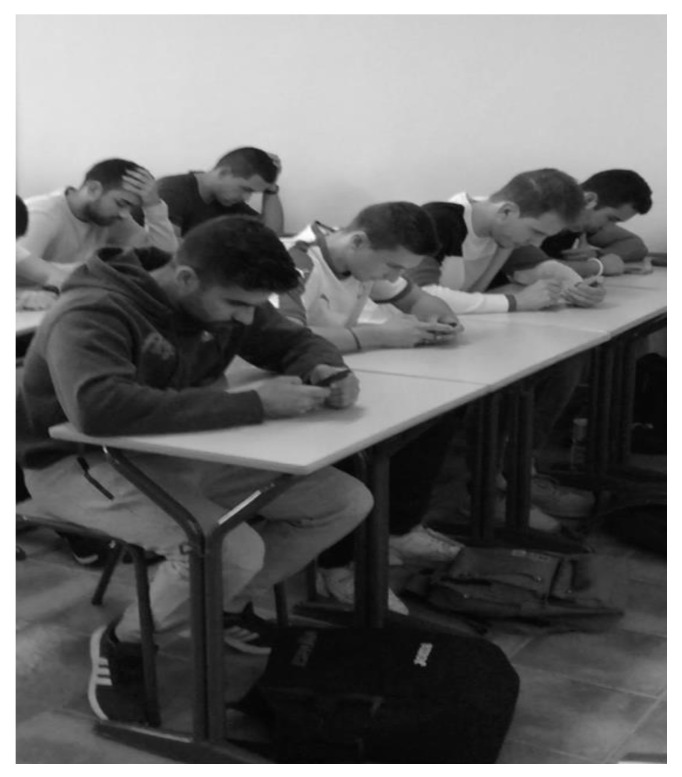
Experimental Set. Student performing the PVT (see text for full description).

The task duration in both preintervention and postintervention was a 10-minute test [42]. Students completed the first PVT, and five trials were excluded from the analysis. In addition, these trials were considered as practice in the preintervention for both groups (See Figure 4, for more information).

### 2.5. Data Analysis

For data processing and mean and standard deviation were used. Descriptive statistics were calculated for each variable. For the comparison of samples and to observe statistically significant differences between groups, ABG vs. CG, was used as the between-subjects factor, and time of measurement, baseline vs. eight weeks, as a within-subject factor. We performed a paired-sample *t*-test in body composition characteristics (body weight, BMI) and physiological parameters (RPE). Effect size is indicated with Cohen’s d for *t*-tests [0.2 (small); 0.5 (medium) and >0.8 (large)] and partial eta squared for Fs. In addition, confidence intervals (95%) were calculated.

Analyses of variance (ANOVA) were used to analyze the RTs. Trials with RTs below 100 ms in the experimental group (preintervention = 11.38% and postintervention = 7.79%) were assumed to represent anticipation errors and were discarded from the analysis [18].

Statistically significant effects were further analyzed with paired-sample *t*-tests corrected by Holm-Bonferroni for multiple comparisons. The Greenhouse–Geisser correction was applied when sphericity was violated. Data were analyzed using Statistical software (version 10.0; Statsoft, Inc., Tulsa, OK, USA). For all analyses, significance was accepted at *p* < 0.05.

## 3. Results

### 3.1. Anthropometrical Characteristics

A paired sample *t*-test with body weight between CG (66.79 ± 9.59; CI 95%: 4.68) and ABG (67.40 ± 17.07; CI 95%: 8.38) was not significant [t(21) = 0.05, *p* > 0.05, d = 0.04]. Another *t*-test with BMI between CG (22.90 ± 3.74; CI 95%: 1.25) and ABG (22.41 ± 3.74; CI 95%: 1.83) also was not significant [t(21) = 0.40, *p* > 0.05, d = −0.13]. These results confirmed that there was no statistically significant difference between the groups, therefore, both groups were equal.

### 3.2. Physical Fitness Assessment

The level of physical fitness was assessed by means of the ALPHA-Fitness test battery. A paired sample *t*-test with *Standing broad jump* between ABG (172.71 ± 35.84; CI 95%: 14.28) and CG (177.38 ± 43.96; CI 95%: 21.09) was not significant [t(21) = 0.05, *p* > 0.05, d= −0.09]. Another *t*-test with 4 × 10m speed-agility test between ABG (10.55 ± 2.2; CI 95%: 0.58) and CG (10.98 ± 1.23; CI 95%: 1.05) also was not significant [t(21) = 0.05, *p* > 0.05, d= −0.24]. Finally, a *t*-test with 20-m shuttle run test between ABG (43.91 ± 6.75; CI 95%: 3.41) and CG (43.08 ± 7.25; CI 95%: 2.99) also was not significant [t(21) = 0.05, *p* > 0.05, d= 0.12]. As was the case in anthropometrical characteristics, results confirmed both groups were equal at the start.

### 3.3. Rating of Perceived Exertion (RPE)

A paired sample *t*-test with RPE scale showed higher values in the ABG (16.10 ± 1.21; CI 95%: 0.52) than in CG (6.29 ± 0.42; CI 95%: 0.18) [t(21) = 35,35, *p* < 0.001, d = −10.83]. Previous results confirmed that effort (CG vs. ABG) was different in terms of physical demands.

### 3.4. Psychomotor Vigilance Task

**Figure 5:**
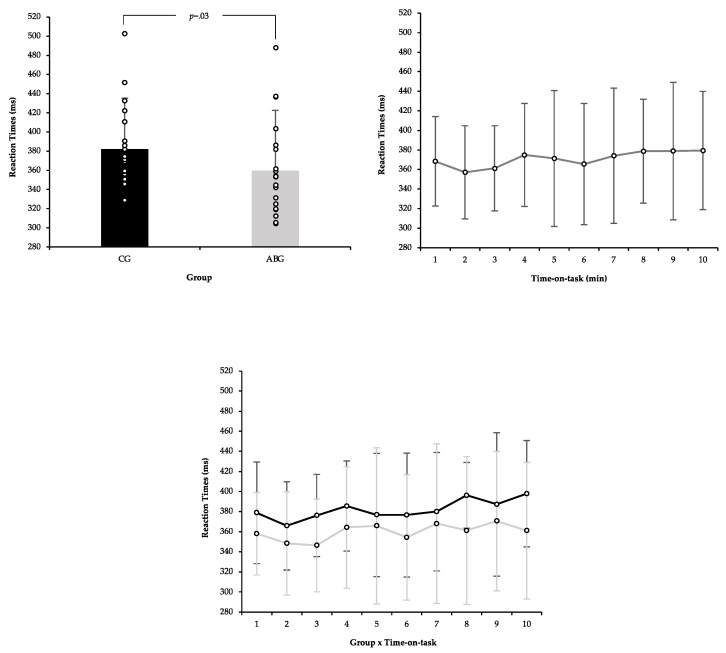
Mean RT (± SE) as a function of Group Condition, time-on-task and Group x time-on-task.

A different analysis of variance of repeated measures (ANOVA) was performed with the average of the participants’ RTs with the groups (CG vs. ABG) and time-on-task (10 min). First, an ANOVA with participants’ mean RT [Pre-CG (380.08 ± 59.41 ms; CI 95%: 17.27) and Pre-ABG (375.97 ± 57.09 ms; CI 95%: 14.56)] and time-on-task, was not significant in any effects or interactions [*F* < 1 in all cases]. Second, an ANOVA with participants’ mean RT [Pre-CG (380.08 ± 59.41 ms; CI 95%: 17.27) and Post-CG (382.05 ± 53.21 ms; CI 95%: 20.33)] also was not significant in any effects or interactions [*F* < 1 in all cases]. Finally, a new ANOVA with participants’ mean RT [(Post-CG (382.05 ± 53.21 ms; CI 95%: 20.33) and Post-ABG (359.76 ± 62.89 ms; CI 95%: 22.91)] revealed a significant main effect of group condition [F = 4.89, *p* = 0.03, η2 = 0.19]. Participants responded faster in the ABG than in the CG. The effect of time-on-task and interaction between the control condition and time-on-task was insignificant (*F* < 1). More information is in Figure 5.

## 4. Discussion

The present study investigated the chronic effects of an eight-week training program on vigilance performance in high school students. The results revealed faster RTs in the experimental group than in the CG. However, the effect of time-on-task and interaction between the control condition and time-on-task was not significant (*F* < 1). Crucially, our results showed a significant main effect of the group with faster RTs in the ABG than in the CG. This result suggests a facilitation effect on vigilance in the ABG and provides support to previous research that showed moderate aerobic exercise had a selective impact on cognitive processing [43,44]. Therefore, the inclusion of uncertainty regarding the appearance of the target in the PVT makes it different from simple RT tasks and provides a reliable instrument to measure vigilance. Thus, in our study, the PE not only improved the nonspecific response speed but rather improved participants’ vigilance.

As previously noted, practicing regular PE has been shown to produce changes to structural and functional levels of the brain [3,44,45]. Chronic PE produces lasting physiological adaptations [46]. Therefore, the body will naturally adjust, finally producing different anthropometric and physiological changes, thus causing an increase in the individual functional level (improved capacity and effectiveness in exercise). Considering the above, the conclusion from the literature is that physical fitness is one of the moderators between the effect of PE and cognitive function [40]. In this respect, we can explain the changes produced by chronic PE in the present experiment, based on the “cardiovascular hypothesis”. Significantly, based on our results, the benefits found for cognitive functions usually associated with the regular practice of PE are moderated by the improvement of physical fitness [3,47,48]. In addition, physiological adaptations at the cardiovascular level, which we suggest occurred due to improvement in vigilance values, are associated with regular PE and have also been associated with adaptation at the brain level, which have been related to improvements in cognitive performance [47,48]. This could be considered a potential limitation of our study since the healthy lifestyle questionnaire and the level of physical fitness of the ALPHA-Fitness test battery were only applied in the preintervention. Consequently, we can only suggest the facilitation effect on vigilance in the ABG.

In the same context, we found interesting studies suggesting that regular aerobic PE is a good stimulus for triggering structural changes at the neural level [3,49] and therefore appears to positively impact cognitive performance [50,51]. Within this specific framework, the new research performed with magnetic resonance techniques [9,14,44,45,47,48] has been linked to adaptations at the brain level, which seem to have a positive impact on cognitive performance. In this respect, the literature revealed that chronic exercise leads to maintenance and neuronal proliferation in different brain areas (especially the hippocampus) and causes the growth of new blood capillaries through the action of brain-derived neurotrophic factor (BDNF) and insulin-like growth type 1 or somatomedin (IGF-1) in the hippocampus, cortex, and cerebellum, which has consequently been shown to have repercussions at the level of cognitive function [52]. Both proteins have shown a permanent increase in their production with the lasting intervention of regular physical exercise [15,53] and could be decisive preventive factors for brain degeneration, long-term enhancers and the development and protein for new neurons.

Finally, regarding the relationship between the chronic practice of PE or the level of physical fitness and general cognitive functioning, it should be noted that practically all of the literature explains the association between these variables based on the premise of the cardiovascular hypothesis, and mainly shows studies in children and older adults. According to this hypothesis, the cognitive function benefits associated with regular exercise are mediated by improving physical fitness. In addition, physiological adaptations attributed to chronic PE have also been linked to adaptations at the brain level, which seem to have a positive impact on cognitive performance [47,48].

Regarding the absence of fitness improvements, such a fact can be determined by the limited volume and intensity of practice [54,55,56]. Some fitness tests are also strength and power-dependent, such as sprinting, jumping and change-of-direction [57,58]. The program provided was based on strength endurance; however, intensity and intention were not controlled, which may cause a bias in the results as intensity may be critical for improvements [59]. Additionally, extra activities performed outside were not controlled, which may constrain the effects of parallel stimulus on the final outcomes.

This study has some limitations. One of the limitations is the absence of a counter-balanced intervention aiming to test different AB effects for the same target group. An additional study limitation is not controlling the extra activities and the effects of baseline levels of students. Baseline levels may play an important role in the progression since being a good or bad responder can be constrained by the starting point and trainability. Despite these limitations, this study provides an important and innovative approach to a micro-dose strategy for improving the quality of life and health of populations. This is one of the few studies dedicated to active break effects in a programmed approach that may help better understand the minimal effective dose that can be applied in students. Future research may compare different micro-doses and intensities while extending the approach to working, elderly and other populations).

## 5. Conclusions

The outcome of the present study suggests that an eight-week PE program based on AB of 16.10 ± 1.21 of the RPE scale improves vigilance performance. The importance of these findings is partly due to the sample of adolescent participants since most previous research has been done on children and adults. In addition, our study highlights a potential finding that locates the basis of dose-response on AB studies. Taken together, the current dataset extends this topic of research and contributes to demonstrating the evidence of the effect of chronic exercise on cognition. It is suggested, however, that future research should systematize greater monitoring of training, not just pre and postintervention. Consequently, another important factor is to analyze the characteristic of ABs (physical exercise, technical exercise, mindfulness, integration in the classroom contents, etc.), as they must be understood in order to assess the best impact on vigilance during the class. In addition, it is recommended that training interventions be carried out for more extended periods of time so that it will be possible to investigate the behavior of vigilance capacity as training time increases. It also contributes to the extant research on cognitive performance during the PA performed in the classroom and opens up exciting avenues for future research.

## Figures and Tables

**Table 1:** Participants’ characteristics (mean ± SD) in the present study.

	Participants		
	CG *n* = 21	ABG *n* = 21	Total *n* = 42
Age (years)	16.52 ± 0.60	16.48 ± 0.60	16.50 ± 0.59
Height (cm)	170.32 ± 7.94	171.80 ± 8.34	171.08 ± 8.07
Weight (kg)	66.79 ± 9.59	67.40 ± 17.07	67.10 ± 13.76
BMI (kg·m^−2^)	22.90 ± 2.44	22.41 ± 3.74	22.64 ± 3.16
Agility test (s)	10.98 ± 1.23	10.55 ± 2.21	10.77 ± 1.78
Standing broad jump (cm)	177.00 ± 0.44	173.00 ±0.36	175.00 ± 0.40
20-m shuttle run test [V02max (mL/kg/min)]	43.08 ± 7.25	43.91 ± 6.75	43.50 ± 6.94
IPAQ-SF (Mets)	712.85 ± 435.40	686.85 ± 402.99	699.85 ± 419.28

Note. CG: Control Group and ABG: Active Break Group. VO2_max_ was estimated with the equation by Legger et al., 1982 [34]: VO2_max_ = 5.857 × velocity (km/h) − 19.45.

## Data Availability

Not applicable.
